# The Importation of Orthopedics

**Published:** 1902-11

**Authors:** 


### EDITORIAL NOTES AND COMMENTS.

The Business office of THE JOURNAL-RECORD is No. 64 Marietta Street.

The Editorial office is 318-319-320 The Prudential.

Address all Business communications to Dr. M. B. Hutchins, Mgr.

Make remittances payable to THE ATLANTA JOURNAL-RECORD OF MEDICINE.

On matters pertaining to the Editorial and Original Communications address Dr. Bernard Wolff, Atlanta.

Reprints of original articles will be furnished at cost price. Requests for the same should always be made *on the manuscript.*

We will present, post-paid, on request, to each contributor of an original article, twenty (20) marked copies of THE JOURNAL-RECORD containing such article.

### THE IMPORTATION OF ORTHOPEDICS.

THE orthopedic faction in this country will have to go 'way back and sit down for quite awhile. The importation of the real thing of the bone-straightening push in that Mecca of American medical come-ons, Vienna, concerned a congenital dislocation of the hip in a young sprigerine of that branch of the American nobility connected with the hog-killing industry. The operation performed was one of great newspaper delicacy, and as the eminent dislocation discourager stretched the kinks out of his back on the completion of his mighty task, he remarked, with a satisfied grin that probably displayed the more or less remote shells of those linked bags of mystery, the fame of which has spread even to Decatur street, where they may be found at any time foregathering with their cisatlantic congener, the hot tamale, "it is finished." In a few short months little Lolita may skip with the insouciance of childhood among the family bull-pens and shambles and mingle her tinkling girlish laughter with the squeal of the assassinated pig "for the first time the hip bones were drawn back into their sockets." In order that the hip bone might not slip out of the socket again, the königlichekaiserlichegeheimrathwündarzt set his first assistant to socket back. There was none of the local orthopedic line-up who could socket back properly. The great Professor Aus Wien was dined by the Chicago Athletic Club in recognition of his manual dexterity and sleight of hand performances and the trained nerve displayed in the presentation of his bill.

The great surgeon was allowed to do his stunt on a few charity cases just to show how dead easy it was if you only knew how, but it is not reported that he gave the same old sigh of relief and said "it is finished." Subsequently he got his kedge fouled in the Illinois Board of Health, but threats of *lese majesté* arising, it was loosened up. "In all the cities arrangements have been made for the Professor to give demonstrations of his method of curing disasters of the hip-joint." We have not heard of any arrangements of the kind having been made here, but a committee must be appointed to round up a few hip-joint disasters in order that the Professor might not get muscle bound before he reached Macon or Savannah.

Hip-joint disasters about here are generally promptly corrected by the doctor nearest to the patient at the time of the disaster, and do not as a rule last long enough to have assistance called in from Vienna (Austria, not Dooly county). Should arrangements not be made the Professor's address ought to be taken down in case consultation be needed in a delicate socket case.

It is a fortunate thing that American doctors have not that sensitiveness to small things (*empfindlichkeit zu kleinigkeiten* as Bismarck phrased it) that marks the German mind, and which had such a beautiful crimp put in it at the time that Sir Morell Mackenzie was sent to see Frederick's cancer of the throat and which nearly threw Berlin into strong spasms.

It would be proper in return courtesy for the present episode for the Germans and Austrians to silence their prejudices against American meat exports. It might influence some other rich American meat market man to send over again for a doctor when there was sickness in the family.

This episode will further teach a lesson to the European "Professors." Hereafter they should keep an emergency grip ready packed, for at any moment some one of them may be called over to remove impacted cerumen from the ear of an Astorbilt, or to apply forceps to some distressed and unwary member of the 400 whose perineum protests against the disgrace of child-bearing and disdainfully resists domestic assistance, and will yield only to the magic of an imported "Open Sesame."

---

### RESOLUTIONS ON THE DEATH OF DR. GILMAN P. ROBINSON, PRESENTED TO THE ATLANTA SOCIETY OF MEDICINE.

WHEREAS, In the dispensation of Providence, death has removed from among us Dr. Gilman P. Robinson, a member of this association and an honored member of the medical profession of the city of Atlanta; therefore, be it

*Resolved*, That in the death of Dr. Robinson this society has lost a member who represented to a high degree the culture and ability which should render our profession worthy of respect and confidence, and which should elevate it to the plane which a learned profession should occupy in the esteem of the public.

*Resolved*, That we deplore the loss of a worthy member who, but for the hindrances placed in his path by ill-health, would have occupied a worthy place in this society and in the medical profession of this city.

*Resolved*, That this society extends to the bereaved wife of the deceased its fullest sympathy and commiseration in this hour of her deep sorrow.

*Resolved*, That these resolutions be published in THE ATLANTA JOURNAL-RECORD OF MEDICINE and a copy be sent to the bereaved wife of our late associate.

V. O. HARDON,
J. C. OLMSTED,
BERNARD WOLFF

